# Identifying obesity/overweight status in children and adolescents; A cross-sectional medical record review of physicians’ weight screening practice in outpatient clinics, Saudi Arabia

**DOI:** 10.1371/journal.pone.0215697

**Published:** 2019-04-25

**Authors:** Maliha Nasim, Mohammed Aldamry, Aamir Omair, Fadia AlBuhairan

**Affiliations:** 1 Department of Population Health, King Abdullah International Medical Research Center/ King Saud bin Abdulaziz University for Health Sciences, Riyadh, Saudi Arabia; 2 Department of Medical Education, King Saud bin Abdulaziz University for Health Sciences, Riyadh, Saudi Arabia; 3 Department of Pediatrics and Adolescent Medicine, Aldara Hospital and Medical Center, Riyadh, Saudi Arabia; 4 John Hopkins Bloomberg School of Public Health, Baltimore, Maryland, United States of America; University at Buffalo, UNITED STATES

## Abstract

**Background:**

BMI is a feasible and recommended measure for overweight and obesity screening in children and adolescents. The study aimed to determine how often physicians correctly identified obesity/ overweight status in children and adolescents by using BMI percentile charts.

**Methods:**

This retrospective cross-sectional study reviewed the paper medical records of children and adolescents (6–14 years) who visited family medicine and pediatric outpatient clinics (Jan-June 2012) in a medical city in Riyadh. Investigators calculated BMI percentiles (using height, weight, age and gender data retrieved from the records) in order to identify patient weight status. Physician documentation of obesity/overweight diagnoses in patient problem lists were cross checked against their BMI percentile to assess the accuracy of physicians’ identification of weight status. The recommended management plan for identified patients was also recorded.

**Results:**

A total of 481 charts were reviewed, 213 (44%) children were seen by family medicine physicians and 268 (56%) by pediatricians. The sample was equally distributed by gender. Height was undocumented for 13% (71) of visiting patients. Eighteen percent of patients (86) were classified as overweight (35)/obese (51) according to age and sex adjusted BMI percentile. Physicians’ correctly identified and documented weight status in 20% of overweight/obese patients: 17 out of 86 subjects. Weight status identification was higher among pediatricians—25% as compared to family medicine physicians—10% [p = 0.08]. Dietary referral was the most common management plan for the identified children. Physicians were more likely to identify obese children {≥95^th^} compared to overweight {≥85^th^ - 95^th^} children. Subjects whose BMI for age classified them into the highest BMI percentile category {≥95^th^} were more likely to be correctly identified (29%) compared to those classified within {≥85^th^ - 95^th^} category—6% [p = 0.007].

**Conclusion:**

Physician identification of obesity/ overweight status for children and adolescents was low, irrespective of their specialty, and despite the condition being prevalent in the sample. Future research that concentrates on interventions that may improve documentation of obesity/overweight diagnoses and parameters needed for BMI indices would be beneficial.

## Introduction

Overweight and obesity levels in childhood and adolescence is a global public health concern.[1, 2] Worldwide prevalence rates of overweight/obesity have risen considerably in children and adolescents in both developed and developing countries.[3] Curtailing the rise in obesity is prioritized in the WHO’s global action plan on prevention and control of NCDs 2013–2020 and in a recent position paper for the society of adolescent health addressing prevention and treatment of adolescent obesity.[4, 5] There is overwhelming evidence that overweight/obese children and adolescents face social, psychological and physical problems as a consequence of their weight. Obese/overweight children are more likely to suffer from anxiety and depression, be bullied, face discrimination, and report low self-esteem compared to healthy weight counterparts.[6, 7] Furthermore, overweight and obesity has been linked to poor health outcomes and negatively impacts quality of life. It also contributes to populations’ burden of disease by impacting all cause morbidity, mortality, and health related quality of life.[3, 8] Obese children and adolescents are more likely to be obese adults and have more severe disease risk factors than individuals of healthy weight in their youth.[9, 10]

There are several levels to obesity prevention among children and adolescents. Promoting healthy eating, physical activity and limiting sedentary activity are examples of primary prevention strategies. Secondary prevention includes early detection of obesity through BMI monitoring in children and youth. These secondary prevention measures are carried out at primary care practices and community public health clinics by health care providers.[11] In fact, physicians are recommended to screen children six years and older for obesity and to refer or to provide behavioral interventions to help them reduce their weight, for those found to have BMIs in overweight/obese categories.[11] The use of age and sex adjusted BMI percentiles is the recommended method for screening overweight and obesity in children and adolescents, because of its convenience, reliability and known association with adult obesity. Despite being one of the easiest conditions to investigate, identification of overweight/obesity remains one of the most underdiagnosed and undertreated conditions.[12, 13] Furthermore, in clinical practice the documentation rate of overweight/obesity among physicians’ remains very low.[14–22] Studies to date have explored the identification through documentation of obesity in different clinical settings including inpatient settings, ambulatory care and amongst trainees.[14, 18, 22] The reasons why physicians fail to identify overweight/obesity in the notes are unclear; however, patients with higher BMI s are more likely to have weight status documented by physicians than those with lower BMIs.[17, 23] The introduction of electronic medical records has done little to improve the situation.[23]

Obesity among children and adolescents in Saudi Arabia is a significant problem. A recent systematic review concluded that it is rising “at an alarming rate”.[24] In 2010, national overall prevalence rates of overweight (23.1%), obesity (9.3%) and severe obesity (2%) were identified among children/adolescents.[25] Another national study reported the prevalence of overweight (15.9%) and obesity (14.1%) in adolescents.[26] In Saudi Arabia, to date, we are unaware of any studies that have assessed physician practices in identifying overweight and obesity status among children and adolescents. Therefore, in building the knowledge base and exploring whether patterns of identification in the rest of the world mirror those within Saudi Arabia, this study hopes to help improve and support achieving quality health care provision for overweight/obese children and adolescents. This study aimed to explore physicians’ practices with regards to identifying and managing overweight/obese children and adolescents in the ambulatory care setting in a tertiary care hospital in Riyadh, Saudi Arabia. It also compared such practices between primary care providers and pediatricians.

## Methods

### Study design and participants

This was a cross-sectional survey based on a retrospective paper medical record review. All children and adolescents aged 6–14 years who visited either the primary care clinic (Kashmalaan) or the General Pediatrics Clinic (at KAMC-R) during January-June 2012 were included in the study. Age criteria was selected on the basis of the United States Preventative Services Taskforce recommendation that obesity screening begin at six years.[27] The age of 14 years was selected as a cutoff point because the upper age limit for Pediatric clinics is 14 years (patients >14 years are seen in adult clinics) in the medical city. All medical records of patients aged 6–14 years were eligible for study inclusion except those that had height or weight data missing from their clinic visit during the study period.

Sample size for this study was estimated at 454 medical records (in total) (227 records, from each site) based on an estimated proportion of 18% for obese/overweight children (28) with a margin of error of 5% at the 95% confidence level. After consulting with a statistician it was recommended that we review 600 charts (300 each from the pediatrics clinic and the primary care center)to account for any missing data. Due to logistical constraints we were however only able to consecutively review 552 medical records in total. The medical records of patients that had either height or weight data missing from the clinic visit were excluded. A total of 481 (87%) patient medical records were therefore included in the final analysis.

### Study setting

This study was conducted at King Abdul Aziz Medical City, Riyadh, Saudi Arabia (KAMC-R), which is part of the Ministry of National Guard- Health Affairs (MNGHA). At KAMC-R, there is a tertiary care hospital with a capacity of over 1000 beds. A general pediatrics clinic, within the hospital, serves approximately 4000 children and adolescents per month. Across the street, there is a primary care center (Kashmalaan), which is also part of the MNGHA network and provides health services to individuals of all ages. The health care to children and adolescents is provided by primary care physicians (general practitioners, family medicine physicians). Height and weight measurements are recorded routinely by nursing staff at every clinic visit at both sites regardless of physical presenting complaint. Medical records of patients (6-14years) would only contain growth charts if there is a clinical suspicion of growth problem that requires monitoring.

### Data collection: Variables selected for the study

The patient medical records were reviewed by two investigators and the following data was collected: demographics (age, gender), site of physician consultation (primary care center/pediatric clinic), physical measurements (height, weight) as well as any documented medical diagnoses for each patient.

Physician identification of overweight/obese patients was assessed through written documentation of the diagnosis in the patient notes. This variable was coded “Yes” if overweight or obesity was clearly documented in the medical records, and coded “No” where it was not. Course of physician action taken once a diagnosis of overweight or obesity was identified was also reviewed from the records. This data was classified according to the following nine physician response categories; nutritional advice; exercise, both nutritional advice and exercise; referral to dietician; referral to endocrinologist; referral to another specialist, laboratory testing, follow up, and any other physician actions. Each variable was coded Yes/No according to whether or not this information was documented in the medical records.

### BMI classification

As the medical records for patients were paper based, the BMI for each patient was calculated by the investigator and documented. BMI percentile was thereafter identified using CDC BMI for age percentile growth charts for gender and age.[28] Patients were categorized by their percentile ranges into underweight (<5^th^ percentile), healthy weight (5^th -^ 84^th^ percentile), overweight (85^th^ - 94^th^ percentile) and obese (≥95^th^ percentile).[28]

### Data analysis

Data was entered and analyzed using Stata statistical software; (StataCorp. 2017. Release 15. College Station, TX: StataCorp LLC). The categorical variables were presented as frequencies and percentages. The outcome of interest was correct identification of overweight/obesity status by physicians based on BMI calculations and BMI charting on CDC age, gender specific growth charts. The covariates studied included clinic site (pediatric vs. primary care), gender, age category, and BMI percentile group. Chi square test was used to determine any association between the covariates and the outcome variable. A p-value of <0.05 was considered to show a statistically significant association.

Approval from the Institutional Review Board (IRB) at the King Abdullah International Medical Research Center was obtained prior to conducting the study.

## Results

### Sample characteristics

In total 552 medical charts were reviewed of patients attending primary health care centers or pediatrics clinics. However height was undocumented for 71 patients, so they were excluded; a total of 481 (87%) patient medical records were therefore included in the final analysis.

Table 1 displays the demographic and weight distribution of patients according to BMI percentile among the sample. The male: female gender distribution in the sample was equal. Children aged 6 to <10 years made up the majority of the sample; n = 281 (58%). The proportionate distribution of patients was higher from hospital pediatric clinics; 268 (56%) compared to primary care-family medicine clinics where 213 (44%) patients were seen.

**Table 1:** Characteristics of the study subjects (N = 481).

	n	(%)
**Age Group**		
Children < 10 yrs	281	58%
Adolescents ≥ 10 to 14 yrs	200	42%
**Gender**		
Male	241	50%
Female	240	50%
**Site where patient was seen**		
Primary Care-Family Medicine clinic	213	44%
Hospital Pediatrics clinic	268	56%
**Body Mass Index Percentile**		
<5th centile	97	20%
5 to <85th centile	298	62%
≥85th to <95th centile	35	7%
≥95th centile	51	11%

Table 1 shows that 86 children were classified as overweight/obese based on BMI percentiles ≥85^th^ centile on the BMI chart. The prevalence of overweight/obesity was found to be 18% (95% CI: 14.5%, 21.5%), with a higher distribution of patients in the obese percentile of ≥95^th^ centile (11%) as compared to the overweight group of ≥85^th^ to <95^th^ centile (7%); there were 97 (20%) children in the sample who were underweight (<5^th^ centile).

Physicians correctly identified the overweight/obese weight status for (20%) of the sample, 17 out of 86 subjects; Physicians did not identify the weight status of (71%), 33 out of 35 of patients who were classified as obese (according to BMI percentile) and (94%) 36 out of 51 of patients classified as overweight (according to BMI percentile). Among the 17 (20%) of patients whose weight status was correctly identified the most common management plan recommended was referral to a dietician (9 cases), while three patients who were overweight/ obese, had their weight status alone documented in their notes without any further action proposed by their physician (Table 2).

**Table 2:** Physician identification and management of overweight/obese status patients.

Physician identified overweight/obese patients (N = 86)	n	(%)
Yes	17	20%
No	69	80%
**Physician response to** identifying an overweight/obese patient (N = 17*)		
Refer to Dietician	9	53%
Refer to Endocrinologist	2	12%
Referral to Other	1	6%
Documentation only of Diagnosis	3	18%
Nutritional Advice	2	12%
Laboratory Test & Follow-up	1	6%

* One child was referred to both Dietician & Endocrinologist

Table 3 shows the distribution of overweight/obese status by age, gender, and site of visit. It was seen that there was no significant difference in the distribution by age group (p = 0.21) and gender (p = 0.98). There was a borderline significance in the site of visit with slightly more overweight/obese children presenting to the hospital pediatrics clinic (21%) as compared to the primary care- family medicine clinics (15%) (p = 0.09).

**Table 3:** Distribution of overweight/obese status patients by covariates.

		Overweight / Obese status (≥ 85^th^ centile)		
	n	Yes (n = 86)	No (n = 395)	p-value*
**Age**				
Children (<10 yrs)	**281**	45 (16%)	236 (84%)	0.21
Adolescents (≥10yrs)	**200**	41 (20.5%)	159 (79.5%)	
**Gender**				
Male	**241**	43 (18%)	198 (82%)	0.98
Female	**240**	43 (18%)	197 (82%)	
**Site**				
Primary care	**213**	31 (15%)	182 (85%)	0.09
Pediatric	**268**	55 (21%)	213 (79%)	

*After applying a Bonferroni correction on α ≤ 0.05, statistical significance is α ≤ 0.017

**Fig 1:**
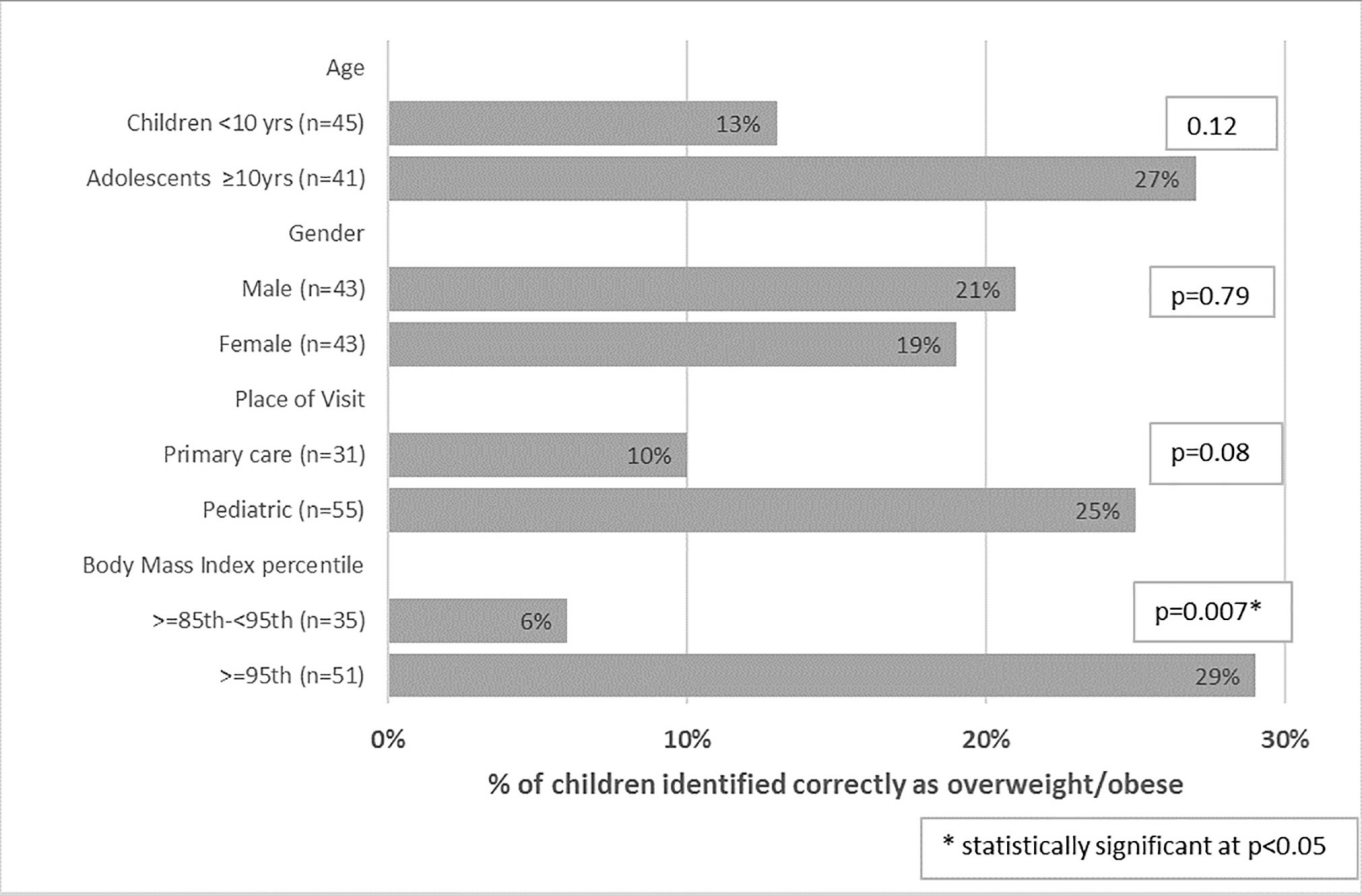
Physician identification of overweight/obese status of patients by covariates (N = 86). *After applying a Bonferroni correction on α ≤ 0.05, statistical significance is α ≤ 0.013.

Fig 1 presents the effect of covariates on physician identification of overweight/obese status of patients. Obese children with the highest BMI percentile ≥95th were more likely to be correctly identified by physicians as compared to patients whose BMI fell in the ≥85^th^ to <95^th^ centile (p = 0.007). Also pediatricians had a borderline significance (p = 0.08) in identifying a higher proportion (25%) of overweight/obese children as compared to family medicine physicians (10%).

## Discussion

Early identification and referral by physicians to behavioral interventions that improve weight status has been recommended as a critical step in the pathway to curtailing the rise in overweight and obesity in children and adolescents. Physician documentation of overweight or obese status in the medical records of children and adolescents through use of BMI screening is essential to identifying cases who are affected or at high risk of being affected by obesity. Furthermore, documentation of overweight/obese status on patients’ problem lists has been shown to increase the rate at which health providers address obesity in future patient visits.[29] The current study showed that physicians are poor at identifying/documenting cases of children and adolescents who are overweight/ obese. Furthermore, children and adolescents with BMIs ≥95th percentile were more likely to be identified by their physicians as compared to patients who fell into the overweight category.

Several interdependent steps are involved in identifying and documenting BMI and overweight/obese status; taking a patient’s height and weight measurements, physician’s clinical suspicion of obesity, BMI calculation, plotting these values on growth charts, discussing diagnosis with patient and documenting it in the medical notes. A failure in any one of these steps can be a cause for poor rates of Identification of overweight /obese status. Assessing the causes for poor physician identification of overweight/obese was beyond the study scope and design; however, we can hypothesize a number of possible contributing factors.

Despite height and weight being routinely recorded on growth charts for all children and adolescents included in the analysis; BMI is not routinely documented in the paper based medical records. Infrequent documentation of BMI in children and adolescents by pediatric physicians has been reported in a handful of studies [17, 20] with documentation rates ranging between 3.0–5.5%. BMI documentation has been found to increase documentation of overweight/obesity [30–32] and national bodies in the United States (US) like the US preventative services task force recommend using BMI to screen for obesity in all children and adolescents over the age of six years. [27] Physician review of patient BMI is a random and unplanned occurrence. In our hospital, physicians are expected to review the BMI of their patients once every year. However, this is not a scheduled event, nor is the responsibility delegated to a single health care provider or setting. Furthermore, quality processes of monitoring and checking documentation of BMI are lacking. Belbins’ studies of teamwork have shown that teams perform best when employees are given clear responsibilities, and responsibilities are known to senior management.[33] Similarly, the importance of clearly defining employee tasks responsibilities and roles within a team and holding team members accountable is widely acknowledged as best management practices to increase performance and productivity within team settings.[34] Delegation of screening for BMI in patients, plotting on CDC charts at the same time growth and weight are recorded and flagging suspected cases may improve BMI documentation and hence early identification of obesity in children and adolescents. Weight status is unlikely to be addressed by physicians unless the patient comes with this as a presenting complaint. Modification of the diagnosis template in the medical records that mandates physicians routinely review and document weight classification as part of their clinical encounter has been shown to be an effective measure in improving physicians documentation of overweight/ obesity status among children. [35]

The study found that there were a slightly higher proportion of overweight/obese children visiting the Pediatrics clinic as compared to the family medicine clinic. Also the pediatricians were borderline more likely to identify overweight/obese children as compared to the family medicine physicians. This finding supports findings from a similar study where pediatrician and primary care physicians were compared in their prevalence of using BMI for age to screen for obesity.[36] Pediatricians’ prevalence of using BMI at well child visits was 50% compared to primary care physicians’ use of 22%. [37] Reasons why pediatricians are better in identifying overweight/obese patients compared to general practice physicians are unknown. Possible explanations may include pediatricians’ exposure to children & adolescents with BMIs in the higher centiles may be greater than those of primary care physicians. This is reflected in our results which show proportionately more children & adolescents with BMIs in the overweight/obese category were attending pediatric clinic compared to those seen by primary care physicians. Children and adolescents with higher BMIs may trigger greater clinical suspicion (based on weight-based presentation of co morbidities and physical appearance) that would trigger further investigation of BMI. One study found that children with a BMI ≥95^th^ centile for age were 10.7 times more likely to have their BMI documented for age. [17] It has also been reported that pediatricians are more confident than primary care practitioners in discussing issues of BMI with their patients. [37] Pediatricians’ confidence in being able to discuss BMI with their patients may be linked to their higher prevalence of documenting the diagnosis in the medical record. Furthermore, pediatricians’ documentation rates of diagnoses may also be linked to their better ability to refer overweight/obese patients for management compared to primary care practitioners. Specialized obesity prevention and treatment services like weight control clinics and dieticians are often located in tertiary care hospitals. Hospital based pediatricians have better accessibility to these services and have advantages in referral of their patients compared to physicians outside the hospital vicinity. In fact one study, reported that pediatricians access to specialized obesity clinics for referral was significantly higher for pediatricians compared to primary care practitioners. [37]

Height and weight measurements are vital prerequisites to BMI calculation, height was missing from 13% of the medical charts reviewed in this study. Therefore, 71 cases of BMI could not be calculated and these subjects were thus excluded from the study. Although this figure is much lower than reported by similar studies (25%), there remains room for improvement.[38] Physician identification of overweight and obese patients would be potentially hindered in cases where patient’s critical height/ weight measurements (needed for BMI calculation) are missing/ unavailable during the patient- physician consultation.

As expected, obese patients were more likely to be correctly identified by physicians compared to overweight patients. This finding supports previous studies, reporting that children with a BMI ≥ 95% for age were more likely to have BMI documented and/or plotted on CDC charts within their medical records. [17, 22] However, in this study, nearly all overweight children and adolescents (94%) were not identified by the physicians they consulted as being overweight. Overweight children and adolescents are known to be at high risk of developing obesity in childhood and as adults. [39, 40] The low percentage of physicians correctly identifying overweight children in this study signifies a missed opportunity to initiate early obesity prevention strategies.

To our knowledge, this is the first study to compare practices in identifying overweight/obese children and adolescents between primary care family medicine physicians and hospital based pediatricians in an outpatient setting. Previous studies have looked at whether hospital physicians at different stages of medical training in their career (medical student, intern, resident, and consultant) differ in their frequency of documenting BMI/overweight/obese status in the notes [17, 18] within inpatient settings. The low documentation rate of overweight/obese status among physicians in our study mirrors those of a similar study which found that physicians regardless of their level of medical training and experience were overall poor at documenting obese/overweight status in the medical records. Out of 300 hospitalized children who according to their BMI were classified as overweight/obese: physicians documented their weight status in only 25 cases (8%) [18].

This study was cross-sectional and based on retrospective review of patient medical records. Therefore, obesity/overweight diagnoses documentation may be underestimated because of issues related to missing data (example height and weight data as seen in our study). Furthermore, the retrospective nature of the study meant that researcher is unable to determine reliability and accuracy of height and weight measurements, moreover measurement bias may be introduced due to differing nursing staff, hospital equipment at different sites etc. It does not give any assessment of physician’s knowledge, perceptions, and attitude towards using BMI to screen for overweight/ obese status or the factors they would themselves identify as potential barriers to documenting obesity/overweight diagnoses within patient charts. It is possible that during consultations, physicians may verbally discuss overweight /obesity status but not document it; these cases would not be captured by this study.

## Conclusion

The outpatient physician clinic consultation is often the first step in parent/child healthcare seeking behavior. This clinical encounter provides an excellent opportunity to screen for physicians to routinely monitor BMI and identify children and adolescents at risk of/ affected by obesity. By doing so, timely appropriate preventative and treatment interventions can be initiated early resulting in better short and long term health outcomes for those affected. The low rates of overweight/obesity diagnosis documentation by physicians (hospital pediatricians and family medicine physicians) in our study indicate that an opportunity for early identification of obesity among this vulnerable population is being missed. Further research that concentrates on looking at factors that may influence physicians’ documentation of obesity and overweight status in patient problem lists are recommended to support efforts to curtail the rising obesity epidemic in this group.

## Supporting information

S1 Codebook [2019_17_Mar_Codebook_submitted to PLOS1]. (DOCX)

S1 Dataset [2019_17_Mar_dataset_for_PLOS1]. (XLS)
