# Correction: Identifying obesity/overweight status in children and adolescents; A cross-sectional medical record review of physicians’ weight screening practice in outpatient clinics, Saudi Arabia

**DOI:** 10.1371/journal.pone.0222853

**Published:** 2019-09-17

**Authors:** Maliha Nasim, Mohammed Aldamry, Aamir Omair, Fadia AlBuhairan

There are errors in the Author Contributions for Fadia AlBuhairan. The correct contributions are: Conceptualization: MN, MA, AO, FA. Project administration: MA, AO, FA. Methodology: MN, FA. Supervision: AO, FA. Writing–review & editing: MN, AO, FA.
