# Supplementary material for: Identifying obesity/overweight status in children and adolescents; A cross-sectional medical record review of physicians’ weight screening practice in outpatient clinics, Saudi Arabia
Source: PLoS One. 2019 Apr 25;14(4):e0215697. doi: 10.1371/journal.pone.0215697 (PMC6483234; doi:10.1371/journal.pone.0215697)
Supplement: S1 Codebook — (DOCX) [file pone.0215697.s001.docx]

| **Variable Name** | **Variable Label** | **Variable Type** | **Valid Value** |
| --- | --- | --- | --- |
| Patient_ seen_ at | Patient seen location | Categorical | Primary care  Pediatrics Clinic |
| Gender | Patient Gender | Categorical | Male  Female |
| Age | Patient Age (Years) | Continuous | From 6 to 14 years |
| Age10yrs | Age categorized according to two subgroups  under 10years  over 10years | Categorical | (6,7,8,9 year olds)  (10,11,12,13,14 year olds) |
| height | Patient height | continuous | Unit: cm |
| weight | Patient weight | continuous | Unit: kg |
| BMIc | Body Mass Index | Continuous | Calculated BMI from height & weight |
| BMI_ Percentile | BMI Percentile | Categorical | <5 = “<5”  5 to <85= “5-<85”  85 to <95= “85-<95”  95+= “>=95” |
| BMC_perecent_481 | BMI percentiles coded as 1-4 | Categorical | 1=“<5”  2=“5-<85”  3=“85-<95”  4=“>=95” |
| Patient_is_ Overweight | Patient is Overweight/Obese | Categorical | Yes  No |
| Obesity_ identified | Overweight\Obesity was identified by physician | Categorical | Yes  No |
| Phy_Actions | Physicians Actions | Categorical | Nutritional Advice  Laboratory Test / Follow-up  Documented only  Refer to Dietician  Refer to Endocrinologist  Refer to Dietician &Endocrinologist  Referral to other |
